# Divergence between confidence and knowledge of endodontists regarding non-odontogenic pain*

**DOI:** 10.1590/1678-7757-2023-0222

**Published:** 2023-10-09

**Authors:** Marcos Dezotti LUIZ, Letycia Accioly Simões COELHO, Rodrigo Ricci VIVAN, Marco Antônio Hungaro DUARTE, Murilo Priori ALCALDE, Paulo César Rodrigues CONTI, Yuri Martins COSTA, Leonardo Rigoldi BONJARDIM

**Affiliations:** 1 Universidade de São Paulo Faculdade de Odontologia de Bauru Departamento de Dentística, Endodontia e Materiais Odontológicos Bauru Brasil Universidade de São Paulo, Faculdade de Odontologia de Bauru, Departamento de Dentística, Endodontia e Materiais Odontológicos, Bauru, Brasil.; 2 Universidade de São Paulo Faculdade de Odontologia de Bauru Departamento de Prótese e Periodontia Bauru Brasil Universidade de São Paulo, Faculdade de Odontologia de Bauru, Departamento de Prótese e Periodontia, Bauru, Brasil.; 3 Universidade de Campinas Faculdade de Odontologia de Piracicaba Departamento de Biociências Piracicaba Brasil Universidade de Campinas, Faculdade de Odontologia de Piracicaba, Departamento de Biociências, Piracicaba, Brasil.; 4 Universidade de São Paulo Faculdade de Odontologia de Bauru Departamento de Ciências Biológicas Bauru Brasil Universidade de São Paulo, Faculdade de Odontologia de Bauru, Departamento de Ciências Biológicas, Bauru, Brasil.

**Keywords:** Knowledge, Facial pain, Endodontics, Continuing Dental Education

## Abstract

This study aimed to assess the self-reported levels of confidence and knowledge related to non-odontogenic pain among a group of Brazilian endodontists.

**Methodology:**

A total of one hundred and forty-six endodontists affiliated with the Brazilian Society of Endodontics participated in the survey. The questionnaire, distributed via email or WhatsApp, contained inquiries designed to gauge self-perceived confidence and knowledge concerning non-odontogenic pain. The practitioners were categorized into four groups based on their self-reported familiarity with various orofacial pain types, classified as either sufficient or insufficient, and on their engagement in ongoing educational programs related to orofacial pain. Data were analyzed by Chi-Square Test and Fischer’s exact test (p<0.05).

**Results:**

Overall, self-reported confidence about non-odontogenic pain was high, especially for endodontists who considered their knowledge about orofacial pain sufficient, regardless of whether they had (71.1% - 97.8%) or not (35.7% - 96.4%) been continuously involved in education courses on orofacial pain. In general, self-reported knowledge about non-odontogenic pain was insufficient (0% - 42%), except in the question about how they would act in cases of pain that persists beyond the normal healing time after an endodontic procedure (70.6% - 81.9%). In general, endodontists are confident in their diagnosis and treatment of non-odontogenic pain. Nonetheless, this confidence did not correlate with a commensurate knowledge depth of. Thus, specialization courses in endodontics should highly consider training and qualifying these professionals in the diagnosis of non-odontogenic pain.

## Introduction

Orofacial pain affects a considerable portion of the population, with odontogenic pain being not only the most prevalent cause of this type of pain^1,2^ but also the major reason why patients seek a dental office.^3^ Therefore, odontogenic pain is the likely diagnosis in many cases. However, opain may present clinical features that resemble non-odontogenic pain, requiring a careful differential diagnosis and assessment.^4^ Thus, distinguishing between odontogenic and non-odontogenic pain can be challenging in certain situations, potentially complicating treatment planning and implementation.^5,6^

This difficulty may be partly associated with limited information regarding the multiple presentations of orofacial pain, a limit that persists even throughout specialty endodontic training. A recent study evidenced that most dental students and dentists claim to be poorly prepared for the diagnosis and treatment of non-odontogenic pain.^7^ It should be highlighted that, in general, there is insufficient curriculum in orofacial pain training for undergraduate dental students^7,8^ and during graduate courses.^9^ Thus, dentists, orofacial pain specialists, researchers and patients need to combine efforts to successfully address the urgent need for quality orofacial pain education.^9^

Furthermore, orofacial pain conditions are historically not sufficiently characterized, except for temporomandibular disorders (TMD).^10^The first edition of comprehensive and internationally accepted diagnostic and classification criteria for orofacial pain was only published recently.^11^ A recent narrative review offers an overview and a brief explanation of how this classification system^11^ could be used by general practitioners and endodontists.^4^

Few studies investigate dentists’ confidence and knowledge regarding non-odontogenic pain.^7,8^ Moreover, there is also a focus on TMD pain,^12,13^ which represents only one facet of non-odontogenic pain within the orofacial domain. There is a shortage of evidence about the endodontist’s knowledge regarding non-odontogenic pain. Such knowledge is of great importance given the prevalence of orofacial pain complains in the endodontist practice.

Thus, based on the premises that: (1) pain commonly manifests within the dental clinic; (2) pain is a common symptom in many endodontic clinical conditions; (3) it is necessary for the endodontist to be able to differentiate odontogenic from non-odontogenic pain to avoid invasive and iatrogenic dental procedures and; (4) thus far, no study has explored the relationship between endodontists’ confidence and knowledge concerning non-odontogenic pain; the aim of the present study was to evaluate the self-reported endodontists’ confidence and knowledge regarding non-odontogenic orofacial pain. Our *a priori* hypothesis was that confidence and knowledge regarding non-odontogenic pain were not associated among endodontists.

## Methodology

This cross-sectional study was approved by The Human Research Ethics Committee (Protocol No. 40225020.0.0000.5417) in accordance with the Helsinki principles. The sample was composed of endodontists of both sexes who obtained their graduation degrees at courses recognized by the Brazilian Federal Council of Dentistry and registered in the representative association of the area in Brazil (Brazilian Society of Endodontics). All these professionals received, by e-mail or WhatsApp, a link containing the Informed Consent Form (ICF) and a questionnaire.

The questionnaire encompassed a series of multiple-choice queries, designed with the intent of assessing endodontists’ self-reported confidence and knowledge levels concerning non-odontogenic pain. This instrument was adapted and structured based on a pre-existing questionnaire.^7^ The first part of the questionnaire focused on variables such as demographic factors, undergraduate time as a dentist and graduate time as an endodontist. The second part was composed of questions aiming to evaluate the professionals’ self-reported confidence and knowledge regarding non-odontogenic pain. The supplemental file contains the questions that were applied.

This questionnaire was integrated into the online platform Google Forms. A link was generated to facilitate access to the questionnaire, which was subsequently sent to the participants preferably via e-mail and/or WhatsApp. Participants were granted the autonomy to decide whether to engage with the survey, after reading and agreeing to participate in the research by signing an Informed Consent Form (ICF). All data collected were stored within the Google Forms tool, protected by a password. Only authorized researchers had the prerogative to access this dataset.

Two pivotal questions were used to categorize the participants: (1) “How would you define your knowledge about the different types of orofacial pain, excluding odontogenic pain?” and (2) “After you graduated as a dentist, have you been involved in continuing education courses about orofacial pain?”. Based on these questions, the endodontists were categorized into four distinct groups, as outlined below:

Group 1 - those who considered their knowledge as sufficient and had been involved in continuing education courses about orofacial pain;

Group 2 - those who considered their knowledge as sufficient and had not been involved in continuing education courses about orofacial pain;

Group 3 - those who considered their knowledge as insufficient and had been involved in continuing education courses about orofacial pain;

Group 4 - those who considered their knowledge as insufficient and had not been involved in continuing education courses about orofacial pain.

### Statistical analysis

Data were expressed as mean and standard deviation (SD) or percentage, where appropriate. To assess associations between participants who categorized their knowledge about non-odontogenic pain as either sufficient or insufficient and their engagement in continuous education courses about orofacial pain, an initial Chi-square test was employed, resulting in a 4x2 table. In case a significant association was identified, subsequent between-group (2x2) comparisons were conducted using Fischer’s exact test. All statistical analyses were performed using Graph Pad Prism 8. For all analyses, the significance level adopted was 5%.

## Results

In this study, a self-screening questionnaire was sent to all endodontics specialists (n=1,088) registered in the Brazilian Society of Endodontics. In total, 146 completed questionnaires were received, a number that constituted approximately 13.4% of the target population. Among the respondents, 57.5% were female, with an average age of 38.24 years (SD: 9.47). The mean times since graduation and since specialization as an endodontist were, respectively, 15.39 years (SD: 9.69) and 11.10 years (SD: 9.37). A noteworthy 87% reported that the content pertaining to non-odontogenic pain during their undergraduate education was insufficient. However, 50% of the participants considered their personal knowledge about non-odontogenic pain to be sufficient.

Fifty-two percent of the respondents reported not having been continuously involved in continuing education courses about orofacial pain and 48% of the respondents sought knowledge about orofacial pain by attending congresses in the area, reading articles and books, taking online courses and attending lectures (81.50%). However, only 18.50% reported having taken a refresher course or a specialization in orofacial pain. A considerable proportion of participants reported encountering a significant prevalence of patients with orofacial pain complaint in their clinical practice (67.5%).

Table 1 contains the six questions that assessed the endodontists’ self-reported confidence levels regarding non-odontogenic pain. Overall, the endodontists considered themselves sufficiently knowledgeable to diagnose and manage non-odontogenic pain. For those who perceived their understanding of orofacial pain to be sufficient, self-reported confidence levels ranged from 71.1% to 97.8% among those who were engaged in continuous education courses, and from 35.7% to 96.4% among those who were not. Conversely, self-reported confidence was lower for the endodontists who considered their knowledge about orofacial pain as insufficient, regardless of having taken (18.2% - 100%) or not (15.7% - 78.4%) continuing education courses about orofacial pain.

**Table 1:** Endodontists’ self-reported confidence regarding non-odontogenic pain

Variable	Confident	Sufficient		Insufficient		Total	P- value Chi-square Test
		Attended a course	Did not attend a course	Attended a course	Did not attend a course		
Question 1- Do you feel confident to differentiate odontogenic pain (pain of dental origin) from non- odontogenic pain (pain of non-dental origin)?	Yes	41 (91.1%)	24 (85.7%)	9 (40.9%)	28 (54.9%)	102 (69.9%)	p<0.0001*
	No	4 (8.8%)	4 (14.3%)	13 (59.1%)	23 (45.1%)	44 (30.1%)	
	Total	45 (30.8%)	28 (19.2%)	22 (15.1%)	51 (34.9%)	146 (100%)	
Question 2- Do you feel confident in stating that non-odontogenic/non-dental pain can lead to referred/located pain in the dental region?	Yes	44 (97.8%)	27 (96.4%)	22 (100%)	40 (78.4%)	102 (69.9%)	p=0.0013*
	No	1 (2.2%)	1 (3.6%)	0 (0%)	11 (21.6%)	44 (30.1%)	
	Total	45 (30.8%)	28 (19.2%)	22 (15.1%)	51 (34.9%)	146 (100%)	
Question 3- Do you feel confident in diagnosing different non-odontogenic/non-dental pain?	Yes	43 (95.5%)	23 (82.1%)	17 (77.3%)	19 (37.3%)	102 (69.9%)	p<0.0001*
	No	2 (4.4%)	5 (17.9%)	5 (22.7%)	32 (62.7%)	44 (30.1%)	
	Total	45 (30.8%)	45(30.8%)	22 (15.1%)	51 (34.9%)	51 (34.9%)	
Question 4- Do you feel confident in treating different non-odontogenic/non-dental pain?	Yes	32 (71.1%)	10 (35.7%)	4 (18.2%)	8 (15.7%)	102 (69.9%)	p<0.0001*
	No	13 (28.9%)	18 (64.3%)	18 (81.8%)	43 (84.4%)	44 (30.1%)	
	Total	45 (30.8%)	28 (19.2%)	22 (15.1%)	51 (34.9%)	146 (100%)	
Question 5- Do you feel confident in diagnosing pain that persists beyond the normal healing period post endodontic treatment?	Yes	38 (84.4%)	23 (82.1%)	16 (72.7%)	33 (64.7%)	102 (69.9%)	p=0.1171
	No	7 (15.6%)	5 (17.9%)	6 (27.3%)	18 (35.3%)	44 (30.1%)	
	Total	45(30.8%)	28 (19.2%)	22 (15.1%)	51 (34.9%)	146 (100%)	
Question 6- Do you feel confident in treating pain that persists beyond the normal healing period after the endodontic treatment?	Yes	38 (84.4%)	21 (75%)	15 (68.2%)	31 (60.8%)	102 (69.9%)	p=0.0748
	No	7 (15.6%)	7(25%)	7 (31.,8%)	20 (39.,2%)	44 (30.1%)	
	Total	45 (30.8%)	28 (19.2%)	22 (15.1%)	51 (34.9%)	146 (100%)	

* indicates significant difference – Chi-square Test

Statistical analyses revealed significant associations in several instances (p<0.05), underpinning the relationships between different groups (Table 1). Regarding question 1, it was possible to observe that endodontists who described their knowledge about different orofacial pain types as sufficient, regardless of the continuing education, demonstrated higher confidence in discerning dental pain from non-dental pain (Table 2).

**Table 2:** Association between groups considering the self-reported confidence regarding non-odontogenic pain

Association	Question 1	Question 2	Question 3	Question 4
Group 1 x Group 3	p<0.05	Ns	p<0.05	p<0.05
Group 1 x Group 2	Ns	Ns	Ns	p<0.05
Group 1 x Group 4	p<0.05	p<0.05	p<0.05	p<0.05
Group 2 x Group 3	p<0.05	Ns	Ns	Ns
Group 3 x Group 4	Ns	p<0.05	p<0.05	Ns
Group 2 x Group 4	p<0.05	p<0.05	p<0.05	Ns

Fischer’s exact test - Ns: no significant difference

Group 1 - endodontists who considered their knowledge as SUFFICIENT and had been continuously involved in continuing education courses in orofacial pain ; Group 2 - endodontists who considered their knowledge as SUFFICIENT and had not been continuously involved in continuing education courses in orofacial pain ; Group 3 - endodontists who considered their knowledge as INSUFFICIENT and had been continuously involved in continuing education courses in orofacial pain ; Group 4 - endodontists who considered their knowledge as INSUFFICIENT and had not been continuously involved in continuing education courses in orofacial pain.

In contrast, in question 2, endodontists who described their knowledge about different orofacial pain types as insufficient, and who did not take courses for the diagnosis and treatment of orofacial pain, exhibited decreased confidence in asserting that non-odontogenic pain could lead to referred pain in the tooth region (Table 2).

Regarding the confidence in the diagnosis of non-odontogenic pain (question 3), the lowest percentages were observed for those who considered their knowledge about different orofacial pain types as insufficient, and who did not take courses for the diagnosis and treatment of orofacial pain. In the significant associations, the attributes of having sufficient knowledge or consistent engagement in continuing education courses pertaining to orofacial pain played pivotal roles.

Regarding the treatment of non-odontogenic pain (question 4), a significant association was verified for endodontists who described their knowledge on orofacial pain as sufficient and who took courses on the orofacial pain area. In this group, the highest confidence levels were found (71.1%), whereas the other groups exhibited diminished confidence levels (< 40%).

Questions 5 and 6 assessed the confidence of endodontic specialists regarding the persistence of pain beyond the customary healing timeframe following endodontic treatment. The confidence level varied from 75.8 to 84.4% (group 1 and 2) and from 60.8% to 72.7%, (groups 3 and 4), with no significant association among the groups (p>0.05).

Despite the satisfactory self-reported confidence, it was verified that the actual knowledge about non-odontogenic pain was low among endodontists (0% - 42%), regardless of whether they considered they had sufficient knowledge about different orofacial pain types and had taken continuing education courses in the area. The only exception refers to the question about the conduct they would have in cases of pain persisting beyond the customary healing period following endodontic treatment, in which the knowledge level was notably high (70.6% -81.9%).

Considering endodontists’ knowledge regarding non-odontogenic pain (table 3), a significant association among groups was verified only for question 4, which referred to “knowledge about the nomenclature of pain that persists beyond the normal healing time after the endodontic procedure”. This association was most pronounced among those who characterized their familiarity with diverse types of orofacial pain as sufficient, regardless of their participation in orofacial pain courses (Table 4).

**Table 3:** Endodontists’ self-reported knowledge about non-odontogenic pain

Variable	Confident	Sufficient		Insufficient		Total	P- value Chi-square Test
		Attended a course	Did not attend a course	Attended a course	Did not attend a course		
Question 1- In your opinion, which one (s) of the following conditions can cause referred/localized pain in the dental region?	Yes	4 (8.88%)	0 (0.00%)	5 (22.7%)	6 (11.7%)	15 (10.2%)	p=0.0681
	No	41 (91.1%)	28 (100.0%)	17 (77.3%)	45 (88.3%)	131 (89.7%)	
	Total	45 (30.8%)	28 (19.2%)	22 (15.1%)	51 (34.9%)	146 (100%)	
Question 2- What treatment strategies do you use when the patient's orofacial pain complaint is not of dental origin?	Yes	15 (33.3%)	8 (28.5%)	7 (31.8%)	12 (23.5%)	45 (30.8%)	p=0.7432
	No	30 (66.6%)	20 (71.5%)	15 (68.2%)	39 (76.5%)	104 (69.2%)	
	Total	45 (30.8%)	28 (19.2%)	22 (15.1%)	51 (34.9%)	146 (100%)	
Question 3- n your opinion, which of the following signs/symptoms may indicate a persistent pain condition that occurs after endodontic treatment?	Yes	10 (22.2%)	6 (21.4%)	5 (31.8%)	17 (33.3%)	38 (26.0%)	p=0.5350
	No	35 (77.8%)	22 (78.6%)	17 (68.2%)	34 (66.7%)	108 (74.0%)	
	Total	45 (30.8%)	28 (19.2%)	22 (15.1%)	51 (34.9%)	146 (100%)	
Question 4- Would you know how to name this type of pain that persists beyond the normal healing time after the endodontic procedure?	Yes	19 (42.2%)	8 (28.5%)	2 (9.09%)	10 (19.6%)	39 (26.7%)	p=0.0156*
	No	26 (57.8%)	20 (71.5%)	20 (90.9%)	41 (80.4%)	107 (63.3%)	
	Total	45 (30.8%)	28 (19.2%)	22 (15.1%)	51 (34.9%)	146 (100%)	
Question 5- How would you manage pain that persists beyond the normal healing time after the endodontic procedure?	Yes	36 (80.0%)	22 (78.6%)	18 (81.9%)	36 (70.6%)	112 (76.8%)	p=0.6329
	No	9 (20.0%)	6 (21.4%)	4 (18.1%)	15 (29.4%)	34 (23.2%)	
	Total	45(30.8%)	28 (19.2%)	22 (15.1%)	51 (34.9%)	146 (100%)	

* indicates significant difference – Chi-square Association Tes

**Table 4:** Association between groups considering the self-reported knowledge about non-odontogenic pain

Association	Question 5
Group 1 x Group 3	p<0.05
Group 1 x Group 2	Ns
Group 1 x Group 4	p<0.05
Group 2 x Group 3	Ns
Group 3 x Group 4	Ns
Group 2 x Group 4	Ns

Fischer’s exact test; Ns: no significant difference

Group 1 - those who considered their knowledge as SUFFICIENT and had been continuously involved in continuing education courses in orofacial pain ; Group 2 - those who considered their knowledge as SUFFICIENT and had not been continuously involved in continuing education courses in orofacial pain ; Group 3 - those who considered their knowledge as INSUFFICIENT and had been continuously involved in continuing education courses in orofacial pain; Group 4 - those who considered their knowledge as INSUFFICIENT and had not been continuously involved in continuing education courses in orofacial pain.

## Discussion

The endodontic diagnosis has fundamental importance for determining the treatment to be performed and requires, from the professional adequate, knowledge and familiarity with diagnostic criteria and classification.^13^ Therefore, it is important for endodontics specialists to know how to differentiate odontogenic pain from non-odontogenic pain. This study evaluated a group of Brazilian endodontists’ levels of confidence and knowledge regarding non-odontogenic pain and the main findings were: 1) self-reported confidence about non-odontogenic pain was high, especially for endodontists who considered their knowledge about different orofacial pain types as sufficient, regardless of their participation in continuing education courses about orofacial pain; 2) despite the high self-reported confidence, the knowledge about non-odontogenic pain was insufficient, which indicates that the self-assessed knowledge was largely overestimated.

These findings are relevant since, according to the interviewed endodontists, 67.5% of patients cite orofacial pain as the main complaint in the office. Although among the various types of pain in the mouth and face regions, dental-origin pain is the most common diagnosis,^1,2^ it is crucial that endodontists know how to diagnose and differentiate the multiple presentations of orofacial pain, such as TMD pain, trigeminal neuralgia, post-traumatic trigeminal neuropathic pain, among others, in order to avoid iatrogenic or unnecessary therapeutic conducts.^4,9^

It is not surprising that the level of confidence in the diagnosis and treatment of non-odontogenic pain was higher in individuals who considered their knowledge on different orofacial pain types as sufficient and had been involved in continuing education courses on orofacial pain. In contrast to our findings, the level of knowledge about non-odontogenic pain was considered insufficient. Differently from our findings, a previous study reported that only 23% of general dentists affirmed they had “good” or “very good” confidence for diagnosing non-dental orofacial pain.^7^ Thus, we can observe that, although there could be an increase in the confidence of specialists in relation to general practitioners, this does not necessarily translate into a higher knowledge level.

People tend to have overly favorable views on their abilities in many social and intellectual domains.^14^ The high confidence levels and insufficient knowledge observed in this study indicates that individuals have difficulties in recognizing their true skill levels. Thus, a person’s lack of knowledge and skills in a certain area cause them to overestimate their own competence. This phenomenon is reported in the literature as the Dunning-Kruger effect.^14^ These authors were pioneers on demonstrating such association in a series of experiments on abilities in domains such as logical reasoning, humor and grammar.^14^

Moreover, people have difficulty acknowledging their deficiencies for fearing that it can affect them professionally or even because they rarely receive negative criticism in their daily lives.^14,15^ One of the important conclusions of these experiments was that, by developing their skills, individuals also improve their ability to recognize their own limitations and, therefore, can make more accurate self-assessments.

The insufficient knowledge about non-odontogenic pain may, in part, be related to the absence of comprehensive and internationally accepted diagnostic and classification criteria for orofacial pain, since those were only released recently.^11^ Historically, orofacial pain conditions were insufficiently characterized, with the probable exception of TMD. Thus, this lack of scientific agreement and consensus on the main characteristics of orofacial pain may have led to confusion, misconceptions and misclassifications and, therefore, to gaps in the knowledge of diagnosis and treatments.^10^ In order to fill this knowledge gap, the first edition of the International Classification of Orofacial Pain (ICOP)^11^ was recently published, and it may be interesting to follow up the possible educational effects of this classification in future investigations.

Although the diagnosis of most cases of pain complaints within the endodontic clinic can be straightforward and not bring challenge in decision-making, in some cases, misinterpretation of pain origins may lead to misdiagnosis and subsequent iatrogenic treatment.^4^ A suggestion to improve endodontists’ competence in facing cases of non-odontogenic pain would be the implementation a minimum training in orofacial pain, which would benefit students. For instance, the presentation and discussion of current criteria for the diagnosis and classification of these clinical conditions through the dissemination of the ICOP and the explanation on how to use it.^11^ A recent review describes orofacial pain according to the ICOP and how this classification system can assist general practitioners and endodontists differentiate diagnosis of dental and non-dental pain,^4^ which in most cases would help prevent unnecessary and potentially harmful dental planning errors and procedures.^9^

Finally, a worrying result observed in the present study was that 87% of the respondents considered that the content on non-odontogenic pain taught during their undergraduate was insufficient, which was also found in a previous study.^7^ Thus, there is an urgent need to implement a minimum curriculum and training for undergraduate students on orofacial pain, so that they can develop competencies and skills related to diagnosis and treatment under supervised clinical training.^4,13,16^

Some limitations of this present research should be observed: it involved only endodontic specialists who are members of the Brazilian Society of Endodontics, which is not a nationally representative sample of this specialty, thus, the results cannot be generalized. Moreover, the questionnaire did not cover all the necessary knowledge to reach a diagnosis of non-odontogenic pain. However, the purpose of the research was not to identify whether the endodontic specialist felt capable of performing diagnosis and treatment of such conditions, but to identify basic knowledge and, based on that, propose strategies to improve the training of endodontists on orofacial pain, especially to differentiate odontogenic and non-odontogenic pain diagnosis.

## Conclusion

This study indicates that most of the participants consider themselves confident in the diagnosis and treatment of non-odontogenic pain. Nonetheless, such confidence does not correspond to a proportional knowledge level, as evidenced by the identified knowledge gap. Thus, the implementation of training and qualification of these professionals in the diagnosis of non-odontogenic pain are highly recommended and can assure them both safety in clinical decision-making and avoidance of potential iatrogenic and unnecessary dental procedures.
